# Efficacy and safety of rectal nonsteroidal anti-inflammatory drugs for prophylaxis against post-ERCP pancreatitis: a systematic review and meta-analysis

**DOI:** 10.1038/srep46650

**Published:** 2017-04-25

**Authors:** Yi-Chao Hou, Qiang Hu, Jiao Huang, Jing-Yuan Fang, Hua Xiong

**Affiliations:** 1 Division of Gastroenterology and Hepatology, Key Laboratory Gastroenterology and Hepatology, Ministry of Health, State Key Laboratory for Oncogenes and Related Genes, Renji Hospital, School of Medicine, Shanghai Jiao Tong University, Shanghai Institute of Digestive Disease, Shanghai 200001, China

## Abstract

Rectal nonsteroidal anti-inflammatory drugs (NSAIDs) are not commonly used clinically for preventing post-endoscopic retrograde cholangiopancreatography (ERCP) pancreatitis. To evaluate the efficacy and safety of NSAIDs for post-ERCP prophylaxis, we systematically reviewed sixteen randomized controlled trials (involving 6458 patients) that compared rectal NSAIDs with placebo or no treatment for post-ERCP pancreatitis prophylaxis updated to August 2016. GRADE framework was used to assess the quality of evidence. There was “high quality” evidence that rectal NSAIDs were associated with significant reduction in the risk of overall post-ERCP pancreatitis (RR, 0.55; 95% CI, 0.42–0.71). Subgroup analyses demonstrated that diclofenac (RR, 0.41; 95% CI, 0.19–0.90) was probably superior to indomethacin (RR, 0.58; 95% CI, 0.45–0.75), post-ERCP administration (RR, 0.46; 95% CI, 0.24–0.89) was probably superior to pre-ERCP (RR, 0.53; 95% CI, 0.42–0.67), and that mixed-risk population received more benefits (RR, 0.54; 95% CI, 0.33–0.88) than average-risk population (RR, 0.60; 95% CI, 0.41–0.88), but less than high-risk population (RR, 0.41; 95% CI, 0.19–0.91). Moreover, “high quality” evidence showed that rectal NSAIDs were safe when given as a standard dose (RR = 0.80; 95% CI, 0.47–1.36). In conclusion, this meta-analysis revealed that rectal NSAIDs are effective and safe in the prevention of post-ERCP pancreatitis in populations with all levels of risk.

Acute pancreatitis is the most common complication of endoscopic retrograde cholangiopancreatography (ERCP). The reported frequency of post-ERCP pancreatitis varies between 1% and 13.3% in the unselected patients^1^,^2^,^3^, although it may reach 25–39% in certain high risk patients^4^. Because of this, it accounts for considerable morbidity, prolonged hospitalization, increasing healthcare expenditure, severe complications and occasional death^5^,^6^,^7^.

To date, many agents are explored extensively for pharmacological potential against post-ERCP pancreatitis, such as somatostatin^8^, gabexate^9^, ulinastain^10^, nonsteroidal anti-inflammatory drugs (NSAIDs)^11^,^12^ and octreotide^13^. Among these pharmacological agents available for prophylaxis, rectally administered NSAIDs, particularly indomethacin and diclofenac, have exhibited the most promising effect on prevention of post-ERCP pancreatitis^14^. In addition to drug prophylaxis, pancreatic stent placement appears to be effective to lower the risk of post-ERCP pancreatitis in high risk patients^15^. However, increased overall cost of the procedure, technical difficulty, and potential adverse effects, such as pancreatic duct injury, bleeding or infection are main drawbacks of pancreatic stent placement^16^, thereby being less cost-effective in clinical practice for all risk patients. Of note, it has been shown that rectal NSAIDs may reduce the risk of post-ERCP pancreatitis in average risk patients^17^,^18^,^19^, unselected patients^20^,^21^,^22^,^23^,^24^,^25^,^26^ and high risk patients^11^,^12^,^27^,^28^,^29^. On the basis of findings from several previous meta-analyses, rectal NSAIDs (diclofenac or indomethacin) have been recommended by the European Society of Gastrointestinal Endoscopy (ESGE) and Japanese Society of Hepato-Biliary-Pancreatic Surgery (JSHBPS) as guidelines to prevent post-ERCP pancreatitis for all risk patients^30^,^31^. Furthermore, NSAIDs are cheap and simply administered and have a favorable risk profile when given a standard dose (100 mg or 50 mg), making them an attractive option in the prevention of post-ERCP pancreatitis.

So far, however, rectal NSAIDs for post-pancreatitis prophylaxis have not been widely recommended in routine clinical use. Recently, nine new RCTs^17^,^18^,^19^,^20^,^22^,^24^,^26^,^27^,^28^ have been conducted since the last meta-analyses of rectal NSAIDs for post-ERCP pancreatitis prophylaxis and included an additional 4592 patients. To provide clinical practice guidance and a framework for future research in this important area, we therefore conducted a meta-analysis with systematic review of RCTs currently available and evaluated the efficacy and safety of rectal NSAIDs for the prevention of post-ERCP pancreatitis.

## Methods and Materials

### Search strategy

We searched PubMed, EMBASE and the Cochrane Library for studies of NSAIDs in the prevention of post-ERCP pancreatitis updated to August 2016. Key words and/or medical subject heading terms were as follows: (nonsteroidal anti-inflammatory drugs or NSAIDs or indomethacin or diclofenac) AND (post-ERCP pancreatitis or post-endoscopic retrograde cholangiopancreatography pancreatitis or pancreatitis) (see Supplementary Table 1). Reference lists from retrieved articles, review, and meta-analysis were manually searched for additional citations. We also scanned the meeting abstracts presented at Digestive Disease Week, American College of Gastroenterology, United European Gastroenterology Week and Asia-Pacific Digestive Week (2011–2016), and 1 report was found^20^. The search was restricted to adult patients. No language or date restrictions were applied. Disagreement was resolved by joint discussion to reach consensus. When necessary, authors would be contacted for further information.

### Study selection

Articles or abstracts were included if they met the following criteria: (1) the study was a prospective, randomized controlled trial (RCT); (2) compared NSAIDs with placebo or no treatment; (3) examined the efficacy and safety of rectally administered NSAIDs for prevention of post-ERCP pancreatitis; (4) original data not duplicated in another manuscript. Cohort studies, case-control studies, case reports and case series were excluded. The studies focusing on the role of NSAIDs in prevention of post-ERCP pancreatitis using any other routes, such as oral, intraduodenal, intravenous and intramuscular, other than rectal were also excluded. If more than one version of the same study was retrieved, only the most complete or the latest one was used.

### Data extraction and quality assessment

Data extraction was independently performed by 2 reviewers according to the prespecified selection criteria. Disagreement was resolved by discussion between the two authors. The following information from each studies was extracted: first author, publication year, study location, study design, patient characteristics, sample size, intervention approaches (drug form, route, dose and timing), indications and severity criteria. In addition the outcome data of studies, including the number of post-ERCP pancreatitis (any, mild and moderate to severe), amylase concentrations (serum amylase level 2 h or 24 h post-ERCP), the number of hyperamylasemia as well as pain, complications (bleeding, perforation or cholangitis) and deaths were also included. Methodological quality of the included studies was evaluated by using criteria set forth by the Cochrane Collaboration tool for assessing the risk of bias^32^.

### Quality of evidence

The quality of evidence was rated for each summary estimate through the GRADE framework^33^, with main outcomes being ranked based on their relevance to clinical decision assessed as of limited importance, importance, or critical^34^. This approach was also used to rate quality of the evidence for the efficacy and safety of rectal NSAIDs in prevention of post-ERCP pancreatitis as high, moderate, low or very low. RCT began as “high quality” evidence, but can be downgraded by one or two level in accordance with the following criteria: risk of bias, imprecision, inconsistency, indirectness, publication bias. “High quality” represented no more change in current conclusions for effect estimates, whereas “very low quality” suggested that it was very likely to change current conclusions for effect estimates in future^35^.

### Statistical analysis

We performed the meta-analysis in accordance with the recommendations of Cochrane Collaboration^36^. Risk ratio (RR) with the corresponding 95% CI was calculated as dichotomous variable for difference observed between NASIDs group and control group, and weighted mean differences (WMD) were also pooled with 95% CI for continuous variable. Visual inspection of the forest plots was used to identify the statistic heterogeneity, which was further complemented by the *I*^*2*^statistic, a test used to quantify inconsistency across studies resulted from heterogeneity rather than from chance. The statistical heterogeneity was assessed by calculating the Cochran’ *Q* statistic with a significance level of *P < *0.10 or *I*^*2*^ > 50%. Generally, an A *I*^*2*^ of 0–30% represents insignificant heterogeneity, *I*^*2*^ of 30–50% represents mild heterogeneity, *I*^*2*^ of 50–75% represents moderate heterogeneity, and *I*^*2*^ of more than 75% represents substantial heterogeneity. A meta-analysis of intention-to-treat data was performed using the random model Mantel-Haenszel method independent of heterogeneity, which could generate more conservative and reasonable results based on *Q* text or *I*^*2*^ statistic^37^,^38^. For statistically significant treatment effects, the number needed to treat (NNT) to prevent 1 episode of post-ERCP pancreatitis was calculated using the absolute risk reduction (ARR): NNT = 1/ARR. Statistical significance was judged if a *P  * < 0.05, except where otherwise specified.

Primary outcome was the risk of any post-ERCP pancreatitis. Secondary outcomes were the risk of mild or moderate to severe post-ERCP pancreatitis, 2 h or 24 h post-ERCP serum amylase level, the risk of hyperamylasemia as well as post-procedural pain, and adverse events associated with NSAIDs therapy. We also performed several subgroup analyses by grouping the type of NSAIDs (indomethacin or diclofenac), the timing of NSAIDs administration (pre- or post-ERCP), the risk of population (high risk, mixed risk and average risk), mean age of each study (≤60 or >60), pancreatic stent (yes or no). It should be noted that the risk stratification of the patients in included studies was defined based mainly on findings proposed by Masci *et al*.^39^ and Freeman *et al*.^40^ Additionally, we performed multiple sensitivity analyses between sample size (≥300 or <300), setting (single center or multicenter), study format (full text or abstract), and also based on variations in definition of the risk stratification of the patients, diagnostic criterion of pancreatitis as well as criterion of pancreatitis severity. In addition, we repeated the analysis by removing any studies in turn from the overall data to evaluate the influence of any studies on pooled treatment effects.

Publication bias was assessed by visual inspection of funnel plots, and further quantitative analysis by Begg adjusted rank correlation test^41^ and Egger weighted regression method^42^; a *P* value of less than 0.1 was considered representative of statistically significant publication bias. Review Manager (version 5.3.5, Cochrane Collaboration, Oxford, UK) was used for all analyses. All statistical tests were 2-sided.

## Results

### Study characteristics

**Figure 1:**
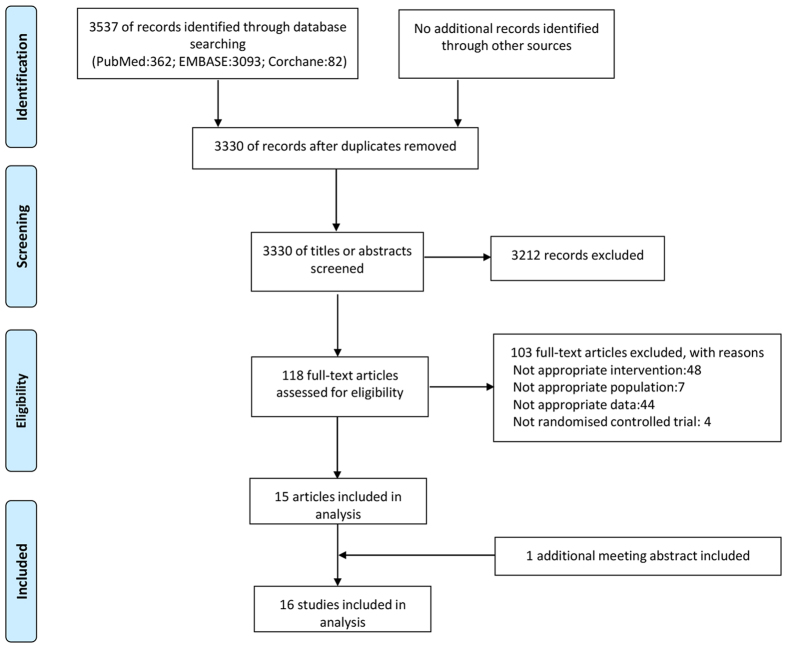
Flowchart of article selection.

The initial search yielded 3537 relevant records of which 3419 were excluded because of duplicate data or based on the screening of titles and abstracts (Fig. 1). The remaining 118 studies were retrieved for full text review. Finally, a total of 15 studies and 1 meeting abstract that included 6438 subjects, including 3226 in treatment group and 3232 in control group, published from 2003 through 2016, were identified in this review^11^,^12^,^17^,^18^,^20^,^21^,^22^,^23^,^24^,^25^,^26^,^27^,^28^,^29^,^43^,^44^. Of the 16 studies, 15 were published in English^11^,^12^,^17^,^18^,^20^,^22^,^23^,^24^,^25^,^26^,^27^,^28^,^29^,^43^,^44^ and 1 in Hungarian^21^. Of these studies, 3 studies were conducted in Hungary^21^,^24^,^43^, 3 in Iran^12^,^17^,^25^, 2 in Mexico^27^,^44^, 2 in America^11^,^22^ and 1 each in China^18^, Sudan^20^, Turkey^26^, Scotland^29^, Japan^23^, Malaysia^28^. Sample sizes ranged from 100^12^,^26^ to 2014^18^, and incidence rate of post-ERCP pancreatitis in control group varied from 4.8 to 26%.

NSAIDs (diclofenac or indomethacin) were all administered rectally in the included studies, either pre-ERCP in 9 studies^17^,^18^,^19^,^21^,^23^,^24^,^25^,^26^,^44^, or post-ERCP in 5 studies^11^,^12^,^27^,^28^,^29^, or during ERCP^22^ in 1 study. NSAIDs 100 mg were used in 15 studies and diclofenac 25 or 50 mg was used in 1 study^23^. Thirteen studies used the definition of post-ERCP pancreatitis based on the consensus criteria^11^,^17^,^18^,^21^,^22^,^23^,^24^,^25^,^26^,^27^,^28^,^43^,^44^, and definition of post-ERCP pancreatitis in an abstract form could not be obtained due to insufficient data^20^. The other two studies defined post-ERCP pancreatitis based on a serum amylase level of greater than fourfold the upper limit of normal in conjunction with abdominal pain^12^,^29^. Of 16 studies, 10 studies^11^,^18^,^21^,^23^,^24^,^25^,^26^,^27^,^28^,^43^ used Cotton criteria to assess the severity of PEP, 2 studies^17^,^44^ used Ranson’s prognostic criteria, 1 study^22^ used the Atlanta symposium guidelines, and the remaining 3 study^12^,^20^,^29^ did not specify the criterion of pancreatitis severity. It is noteworthy that the only criteria for severity that have been validated in the post-ERCP pancreatitis population are the Cotton criteria. No other severity classification scheme has been validated in this population. Pancreatic stent used was mentioned in 7 studies^11^,^12^,^18^,^22^,^27^,^28^,^29^ and varied from 2 to 83%. Of 7 studies, pancreatic stents were placed for therapeutic or prophylactic use, whereas 2 studies^22^,^29^ did not specify its purpose. No statistical significant difference between two groups with or without pancreatic stents. In general, the baseline characteristics of patients and procedures were consistent across two groups in each study with exception of 1 study^23^, in which sex ratio was not comparable between two groups. Basic characteristics of included studies and the main outcome data of each included study are summarized in Tables 1 and 2, respectively.

The risk of bias assessments across studies were described in Supplementary Fig. 1. One meeting abstract was not assigned the risk of bias because of insufficient data^20^.

### Overall post-ERCP pancreatitis

**Figure 2:**
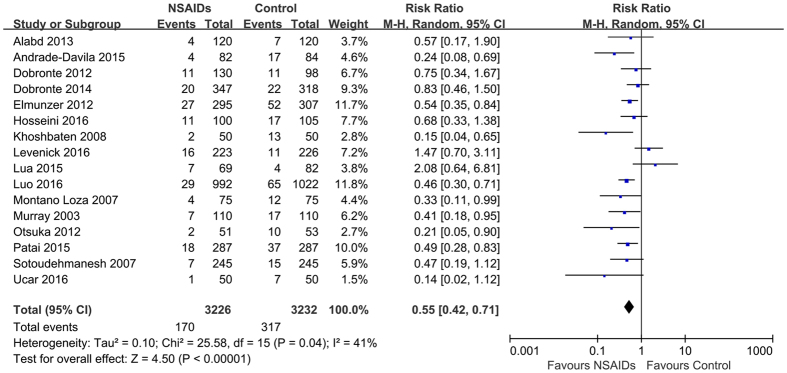
Forest plot showing a significant reduction in the risk of any post-ERCP pancreatitis with rectal NSAIDs therapy. CI, confidence interval; M-H, Mantel-Haenszel; NSAIDs, nonsteroidal anti-inflammatory drugs; ERCP, endoscopic retrograde cholangiopancreatography.

**Figure 3:**
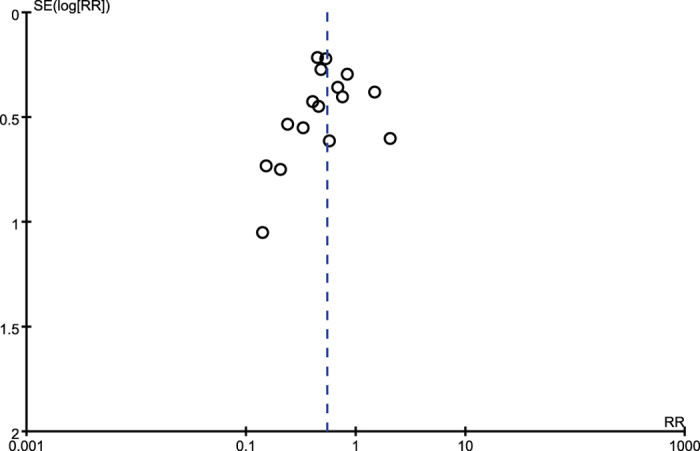
Funnel plot of all included studies did not show asymmetry. Statistical analysis suggested no evidence of publication bias with Begg test and Egger text (*P* = 0.198 and *P* = 0.431, respectively). RR, risk ratio; SE, standard error.

Post-ERCP pancreatitis was documented in 170 of 3226 patients (5.0%) with rectal NSAIDs, compared with 317 of 3232 (9.9%) with no treatment. Pooling showed that rectal NSAIDs were associated with a significant reduction in overall risk of post-ERCP pancreatitis compared with patients with no treatment (RR = 0.55; 95%, 0.42–0.71; *P* < 0.01), with a mild heterogeneity (*P* = 0.04; *I*^*2*^ = 41%) (Fig. 2). The ARR was 5.0% (95% CI, 3–7%). The NNT was 20 (95% CI, 14–33). Sensitivity analysis found that the study proposed by Levenick *et al*.^22^ was the main source of heterogeneity. The prophylactic efficacy of rectal NSAIDs, however, was not affected after removal of this study (RR, 0.51; 95%, 0.41–0.65, *P* < 0.01), with insignificant heterogeneity (*P* = 0.18; *I*^*2*^ = 24%). The quality of evidence of the efficacy of rectal NSAIDs in preventing any post-ERCP pancreatitis was rated “high quality” according to the GRADE framework. No evidence supporting publication bias was identified based on visual inspection of the funnel plot (Fig. 3), Begg test and Egger test (*P* = 0.198 and *P* = 0.431, respectively). Table 3 summarizes the findings and quality assessment for outcomes ranked as critical or important for decision making.

### Subgroup and sensitivity analyses

Subgroup analyses were performed to explore the source of heterogeneity among studies based on key study characteristics and clinical factors. The prophylactic benefit of rectal NSAIDs (indomethacin or diclofenac) on reducing the risk of post-ERCP pancreatitis was consistently found in all of the subgroup analyses. When stratifying studies by drug type, both indomethacin and diclofenac showed a significant efficacy (RR = 0.58; 95% CI, 0.45–0.75; *P* < 0.01 and RR = 0.41; 95% CI, 0.19–0.90; *P* = 0.03, respectively). Pooling estimates showed that rectal diclofenac appeared to be more effective than rectal indomethacin in preventing post-ERCP pancreatitis. Heterogeneity in indomethacin group was insignificant (*I*^*2* ^  = 34; *P* = 0.13), however, heterogeneity in the former group was moderate (*I*^*2 *^  = 55; *P* = 0.05). After removal of the source of heterogeneity,^28^ advantages contributing to the latter group did not change (RR = 0.32; 95%, 0.19–0.56, *P* < 0.01) without heterogeneity (*I*^*2*^ = 0; *P* = 0.52). When stratifying subgroups by timing of administration, rectal NSAIDs administered post-ERCP (RR = 0.46; 95% CI, 0.24–0.89, *P* = 0.02) were more effective than those administered pre-ERCP (RR = 0.53; 95% CI, 0.42–0.67, *P* < 0.01). Heterogeneity was moderate in former subgroup (*I*^*2*^ = 61; *P* = 0.03). After exclusion of the source of heterogeneity^28^, NSAIDs administered rectally post-ERCP were still superior to administered rectally pre-ERCP (RR = 0.39; 95% CI, 0.24–0.63, *P* < 0.01), with insignificant heterogeneity (*I*^*2*^ = 30%; *P* = 0.23).

After stratification according to different risk population, it was noted that rectal NSAIDs were most effective in high-risk population (RR = 0.41; 95% CI, 0.19–0.91, *P* = 0.03). More benefits could be observed in mixed-risk population (RR = 0.54, 95% CI, 0.33–0.88, *P* = 0.01) compared with average-risk population (RR = 0.60; 95% CI, 0.41–0.88, *P* < 0.01). Moderate heterogeneity (*I*^*2* = ^61%; *P* = 0.02) and mild heterogeneity (*I*^*2* = ^49%; *P* = 0.07) separately existed in high-risk and mixed-risk subgroup, although no significant heterogeneity was in average-risk subgroup (*I*^*2*^ = 28%; *P* = 0.25). When removing the source of heterogeneity^28^, the prophylactic efficacy of rectal NSAIDs was not affected in high- or mixed-population (RR = 0.31; 95% CI, 0.16–0.61, *P* < 0.01 or RR = 0.46; 95% CI, 0.33–0.66, *P* < 0.01). As younger age was a definite risk factor for post-ERCP pancreatitis^40^, we therefore take a mean age of 60 years in treatment group as the cutoff point to stratify the studies. It was noted that rectal NSAIDs were more effective for patients with a mean age ≤ 60 years (RR = 0.46; 95% CI, 0.31–0.69, *P* < 0.01) compared with patients with a mean age > 60 years (RR = 0.64; 95% CI 0.43–0.96, *P* = 0.03). However, heterogeneity in latter subgroup was moderate (*I*^*2*^ = 55%; *P* = 0.04). After removing the source of heterogeneity^22^, advantages contributing to older group still existed. In addition, there was no statistical significant difference between two groups with or without pancreatic stents. The details of subgroup analyses and corresponding sensitivity analyses are presented in Table 4. No material change in results was noted in prespecified sensitivity analyses, and none of individual study had significant influence on the pooled estimates.

### Amylase concentration, hyperamylasemia and pain

Only 5 studies^12^,^25^,^27^,^29^,^44^ and 3 studies^12^,^26^,^29^ provided data on the mean levels of serum amylase 2 h and 24 h post-ERCP, respectively. Pooling showed that rectal NSAIDs significantly reduced the mean levels of serum amylase 2 h (WMD = −78.51; 95% CI, −108.41 to −48.61, P < 0.01) (see Supplementary Fig. S2) and 24 h post-ERCP (WMD = −285.02; 95% CI, −440.22 to −129.83, P < 0.01) (see Supplementary Fig. S3) in comparison with control group. There was moderate heterogeneity in latter (*I*^*2*^ = 55; *P* = 0.11). The quality of evidence for the two outcomes was rated as “high quality” and “low quality”, respectively. Six studies^19^,^23^,^24^,^26^,^27^,^44^ provided data on the risk of hyperamylasemia. The incidence of hyperamylasemia was lower than that in control group (RR = 0.59, 95% CI, 0.36–0.96, *P* = 0.03) (see Supplementary Fig. S4). Substantial heterogeneity was present (*I*^*2*^ = 87; *P* < 0.01). Two studies^23^,^26^ reported 39 cases of post-ERCP pain, 9 (8.9%) in rectal NSAIDs group and 30 (29.1%) in control group. Pooling showed that rectal NSAIDs were associated with significant reduction in the frequency of post-procedural pain (RR = 0.32; 95% CI, 0.14–0.77, *P* = 0.01) (see Supplementary Fig. S5), without evident heterogeneity (*I*^*2*^ = 33; *P* = 0.22). The quality of evidence (GRADE) for these two outcomes was both rated as “moderate quality”.

### Severity of pancreatitis

**Figure 4:**
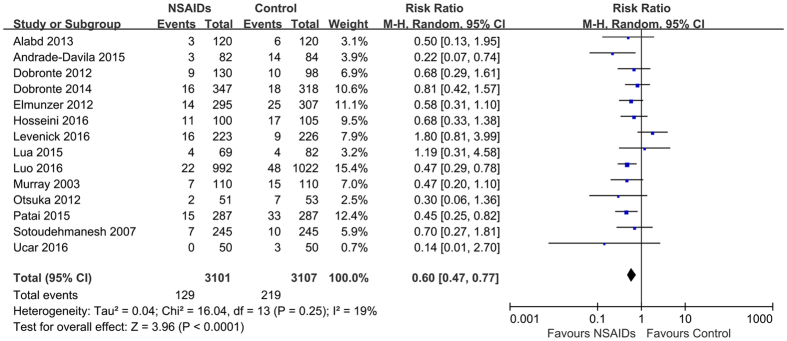
Forest plot showing a significant reduction in the risk of mild post-ERCP pancreatitis with rectal NSAIDs therapy. CI, confidence interval; M-H, Mantel-Haenszel; NSAIDs, nonsteroidal anti-inflammatory drugs; ERCP, endoscopic retrograde cholangiopancreatography.

**Figure 5:**
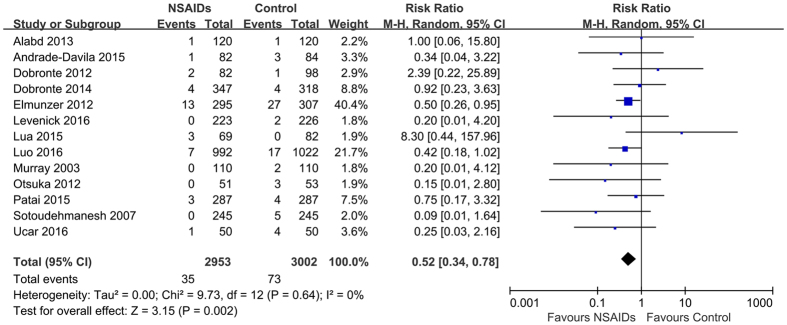
Forest plot showing a significant reduction in the risk of moderate to severe post-ERCP pancreatitis with rectal NSAIDs therapy. CI, confidence interval; M-H, Mantel-Haenszel; NSAIDs, nonsteroidal anti-inflammatory drugs; ERCP, endoscopic retrograde cholangiopancreatography.

Fourteen studies^11^,^17^,^18^,^19^,^20^,^21^,^22^,^23^,^24^,^25^,^26^,^27^,^28^,^29^ and 13 studies^11^,^18^,^19^,^20^,^21^,^22^,^23^,^24^,^25^,^26^,^27^,^28^,^29^ provided data on the severity of post-ERCP pancreatitis, including the risk of mild and moderate to severe post-ERCP pancreatitis, respectively. Fourteen studies reported 348 cases of mild pancreatitis, 129 (4.2%) in the rectal NSAIDs group and 219 (7.0%) in the control group, and 13 studies reported 107 cases of moderate to severe pancreatitis, 35 (1.2%) in the rectal NSAIDs group and 72 (2.4%) in the control group. Pooling showed that rectal NSAIDs were associated with significant reduction in the risk of mild (RR = 0.60; 95% CI, 0.47–0.77, *P* < 0.01) (Fig. 4), and moderate to severe post-ERCP pancreatitis (RR = 0.52; 95% CI, 0.34–0.78, *P* < 0.01) (Fig. 5). No significant heterogeneity was present (*I*^*2*^ = 19; *P* = 0.25 and *I*^*2*^ = 0; *P* = 0.64, respectively). The quality of evidence for this two outcomes was rated as “high quality”.

### Adverse events

**Figure 6:**
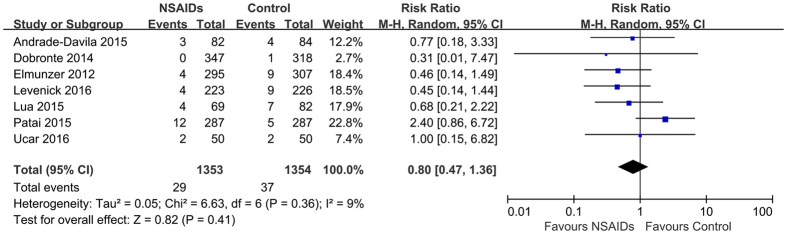
Forest plot showing no statistic differences in adverse events attributable to NSAIDs therapy. CI, confidence interval; M-H, Mantel-Haenszel; NSAIDs, nonsteroidal anti-inflammatory drugs.

Six studies^11^,^22^,^24^,^26^,^27^,^28^ reported the relevant adverse events associated with NSAIDs therapy. Six studies reported 44 cases of bleeding, 22 each in two groups. Pooling showed that rectal NSAIDs did not increase the risk of bleeding (RR = 0.97; 95% CI, 0.49–1.94, *P* = 0.94) with no evident heterogeneity (*I*^*2*^ = 14; *P* = 0.33). Two studies^24^,^28^ reported 2 cases of perforations, both of which occurred in rectal NSAIDs group. Two studies^24^,^28^ reported 10 cases of cholangitis, 4 (1.2%) in the rectal NSAIDs group and 10 (2.9%) in control group. Pooling showed that rectal NSAIDs did not significantly increase the risk of perforation and cholangitis (RR = 3.27; 95% CI, 0.34–31.19, *P* = 0.30 and RR = 0.74; 95% CI, 0.21–2.63, *P* = 0.64, respectively) with no evident heterogeneity (*I*^*2*^ = 0; *P* = 0.94 and *I*^*2*^ = 0; *P* = 0.69, respectively). One study^27^ reported 4 case of anal itching, 2 each in two groups. Two case of acute renal failure (ARF) were reported in 1 study^11^, both of which occurred in the control group. Three studies^19^,^22^,^24^ reported 11 cases of death, 3 (0.3%) in the rectal NSAIDs group and 8 (0.9%) in control group. Pooling showed that there was no statistical significance of rectal NSAIDs in increasing the mortality rate (RR = 0.49; 95% CI, 0.15–1.69, *P* = 0.26), with no heterogeneity (*I*^*2*^ = 0; *P* = 0.58). Generally, an arithmetical trend was noted that rectal NSAIDs may result in a decrease in risk of overall adverse events (RR = 0.80; 95% CI, 0.47–1.36, *P* = 0.41), with no evident heterogeneity (*I*^*2*^ = 9; *P* = 0.36) (Table 3) (Fig. 6). The quality of evidence for all above mentioned outcomes was rated as “high quality”. Other studies, with one exception^18^, did not report relevant complications or deaths potential attributable to the study intervention during the follow-up period.

## Discussion

In this meta-analysis of 16 RCTs that included 6458 patients, there was “high quality” evidence to suggest that rectal NSAIDs were associated with about a 45% decrease in the risk of post-ERCP pancreatitis. The NNT was 20. After stratifying studies according to the severity of post-ERCP pancreatitis, “high quality” evidence showed that a significant reduction was recognized in both mild and moderate to severe post-ERCP pancreatitis (40 and 48%, respectively). Specific analyses for amylase concentration, hyperamylasemia, and pain indicated that there were “high quality”, “low quality”, “moderate quality” and “moderate quality” evidence that rectal NSAIDs could prevent the elevation of serum amylase 2 h or 24 h post-ERCP, lower the risk of hyperamylasemia and relieve the post-procedural pain, respectively. In terms of safety, we also found “high evidence” that no statistical difference in adverse events potentially attributable to rectal NSAIDs therapy was detected, strongly indicating that rectal NSAIDs are safe when given as standard doses (100 mg or 50 mg). Although mild heterogeneity existed in overall post-ERCP pancreatitis, the magnitude or direction of the summary effect remains unchanged in multiple sensitivity analyses.

The present findings are consistent with the current ESGE and JSHBPS guidelines, in which NSAIDs (diclofenac or indomethacin) are widely recommended to administer rectally either pre- or post-endoscopic procedure in preventing post-ERCP pancreatitis for all risk patients^30^,^45^. Recently, however, two studies by Lua *et al*.^28^ and Levenick *et al*.^22^ suggested that there is no significant association between rectal NSAIDs (diclofenac or indomethacin) and the decrease in incidence of post-ERCP pancreatitis among subjects, including high- or average-risk for developing post-ERCP pancreatitis. These findings conflict with these proposed guidelines. Of interest, it is noted that study reported by Levenick *et al*.^22^ was the main sources of heterogeneity in present meta-analysis, in which more than 30% of patients enrolled in this study had previous sphincterotomy and nearly 18% of patients underwent concomitant endoscopic ultrasound or fine needle aspiration, which is a departure from other RCTs included. The mean age of patients included in this trial was 64.9 years, and indomethacin was administrated during the procedure, which separated this trial from other trials. The authors anticipated a sample size of 1398 to detect a 50% reduction in the frequency of post-ERCP pancreatitis from 5% to 2.5% while recruiting consecutive patients with a power of 80% and significance of 0.05. However, the trial was terminated by the Dartmouth Data and Safety Monitoring Committee prior to achieving the estimated number of patients. As a result, only a total of 449 patients were enrolled, leading to only 20% power to demonstrate the hypothesized difference with a significance of 0.05. Though there was a trend of increased incidence of post-ERCP pancreatitis in the indomethacin group, the early termination of the study may have prevented these findings from becoming significant and even reversed if they are taken to completion. The conclusion of this study should not be used to avoid indomethacin in all patients groups as the results of this study are underpowered and are of potential for type 2 error. Although no significant difference in the incidence of post-ERCP pancreatitis was detected between groups with or without rectal diclofenac in high-risk patients, the study reported by Lua *et al*.^28^ lacked statistical power to assess this comparison owning to the small number of high-risk patients included (n = 144) and open-labelled design.

In the present meta-analysis, we explore whether the drug type or administrated timing of rectal NSAIDs had an impact on the outcome. On a subgroup analysis in which the type of NSAIDs (diclofenac or indomethacin) was compared for prevention of pancreatitis, we found that rectal diclofenac seemed more effective than rectal indomethacin for prevention of post-ERCP pancreatitis. As the discovered pharmacological mechanism in animal model, inactivation of phospholipase A2 could result in the inhibition of several important classes of pro-inflammatory lipids, e.g., prostaglandins, leukotrienes and platelet activating factor. In the case of phospholipase A2 inhibition, however, indomethacin demonstrated a stronger inhibitory action compared with diclofenac. As statistics go, rectal diclofenac appeared to be more effective than rectal indomethacin in preventing post-ERCP pancreatitis. However, it should be noted that the number of subjects included in diclofenac group amounted to roughly a seventh of indomethacin group. The confidence interval (0.19–0.90) was quite wide duo to the fact that only 915 subjects were included in 6 studies as opposed to 5543 subjects of indomethacin group in 10 studies, and this should only be considered a very rough estimate of the comparison of rectal diclofenac with rectal indomethacin. To date, there was no larger RCT to perform to compare rectal indomethacin with diclofenac in a head-to-head form to explore any difference between these 2 agents regarding the efficacy in prevention of post-ERCP pancreatitis. Our aforementioned findings differ from the meta-analysis reported by Ding *et al*.^46^, which indicated that indomethacin and diclofenac render the same effectiveness for post-ERCP pancreatitis prophylaxis. It is noted that there were several routes of NSAIDs administration or different doses of diclofenac used in this meta-analysis, which may confounding the outcome to some extent. Three meta-analyses^14^,^47^,^48^ were concordant with our findings, which suggested that efficiency of diclofenac was somehow superior to indomethacin on post-ERCP pancreatitis prophylaxis. One common limitation in these 3 meta-analyses and ours, are that the number of patients included in diclofenac group was far less than that in indomethacin group. Given the above limitation, further RCTs with a sufficient number of patients should focus on comparison between rectal diclofenac and rectal indomethacin in a direct form. Our subgroup analyses also showed that NSAIDs administered rectally post-ERCP seemed to be more effective than those administered pre-ERCP for pancreatitis prophylaxis. After removing the source of heterogeneity, NSAIDs administered rectally post-ERCP still seemed to be more effective in reducing the risk of post-ERCP pancreatitis than those administered pre-ERCP (54% versus 47%). Our findings conflict with the large recent RCT reported by Luo *et al*.^18^, in which routine pre-ERCP rectal indomethacin is superior to post-ERCP rectal indomethacin in lowering the risk of post-ERCP pancreatitis among high-risk patients. The same limitations in present meta-analysis was that the number of patients in post-ERCP subgroup was apparently less than that in comparable group (1088 versus 4530), leading to a lack of adequate statistical performance to verify the real pooled estimates. Hence, further larger RCTs are awaited to determine optimal drug type and timing of administration.

In the past 10 years, numerous meta-analyses have been published to investigate the preventive effect of NSAIDs on post-ERCP pancreatitis; nearly 10 meta-analyses dedicated to assessing efficiency of rectal route on prevention of post-ERCP pancreatitis. Of these meta-analyses, the earlier 2 meta-analyses by Sun, H.L. *et al*.^47^ and Elmunzer, B.J., *et al*.^49^ included lesser RCTs that assessed the effect of rectal NSAIDs on prevention of post-ERCP pancreatitis, which enrolled 1846 and 912 subjects, respectively. The enrolled patients in our meta-analysis were markedly more than those in previous meta-analyses; we also evaluated amylase concentration, the risk of hyperamylasemia and post-procedural pain, relevant complications as well as mortality. Importantly, several subgroup analyses were performed to explore the study heterogeneity based on key study characteristics and clinical factors. Furthermore, multiple sensitivity analyses were also carried out to detect the robustness and validity of overall results. It is almost inevitable that the previously published meta-analyses had several limitations, such as limited sample sizes, lack of precise pooled estimates, poor statistical power and less representative populations. In addition, the quality of evidence for the present outcomes of the review considered critical for clinical decision making was ranked “high” according to the GRADE framework, which could render more precisely and compelling estimates. Recently, one meta-analysis by Inamdar *et al*.^50^, which is currently in press, showed that rectal indomethacin significantly decreases the incidence of post-ERCP in high-risk patients but not in average-risk patients. It should be noted that the authors did not classify unselected patients as independent risk population, and yet just classified included subjects into high- and average-risk patients based on validated patient- and procedure-related independent risk factors^40^. Significantly, Inamdar *et al*. pooled 8 RCTs for their meta-analysis, while the present meta-analysis included a total of 16 RCTs. These extra studies contributed 40.2% of the total random effects weight in the present meta-analysis. Furthermore, Inamdar *et al*. did not assess safety of the drug. In our subgroup analyses by grouping high risk, mixed risk and average risk, which suggested that rectal NSAIDs exhibit more protective benefits in lowering the risk of post-ERCP pancreatitis for high-risk population (59%, *P* < 0.01) compared with mixed- and average- risk population (46 and 40%, *P* < 0.01). To some extent, however, results of present meta-analysis may more accurately reflect the real effects of rectal NSAIDs in preventing post-ERCP pancreatitis for patients categorized by different patient- or procedure-related risk factors due to wider evidence base and sufficient data with greater statistical power. In addition, we did a subgroup analysis on the basis of age, in which mean age 60 in treatment group was deemed to be the cutoff point. The subgroup analysis reveals that rectal NSAIDs may be useful for the reduction of post-ERCP pancreatitis in younger patients (<60) compared with patients over the age of 60. The study reported by Sotoudehmanesh *et al*.^25^ suggested that age lower than 60 years is of significant risk for developing post-ERCP pancreatitis (OR, 2.7; 95% CI 1.04–7.1). In the light of above findings, rectal NSAIDs certainly seemed to be more effective for high risk population, e.g. age <60. Additionally, we find that rectal NSAIDs may lower the serum amylase (2 h and 24 h post-ERCP), the risk of hyperamylasemia and post-procedural pain, indicating that rectal NSAIDs may prevent the elevation of serum amylase, lower the risk of hyperamylasemia, and relieve the pain. The finding conflicts with a meta-analysis reported by Sun *et al*.^47^, in which no significant difference was detected in these values at 24 h following ERCP. One new study^26^ included in our meta-analysis may increase the statistical performance, which accounts for difference between two meta-analyses. It should be noted that substantial heterogeneity was present in pooled estimates for hyperamylasemia. Different definition of hyperamylasemia may accounted for high heterogeneity, such as serum amylase levels >100 IU/mL^23^,^26^,^27^ or 3 times greater than the upper limit of normal^19^,^24^,^44^. It is also important to note that the incidence of post-ERCP pancreatitis in control group varied from 4.8 to 26%, indicating the heterogeneity of the population studied. However, we found that it does not make any significant differences in the risk of overall post-ERCP pancreatitis (RR = 0.57, 95% CI, 0.44–0.73, P < 0.01), mean levels of serum amylase 2 h and 24 h post-ERCP (WMD = −78.51, 95% CI, −108.41 to −48.61, P < 0.01 and WMD = −285.02, 95% CI, −440.22 to −129.83, P < 0.01) when a study by Khoshbaten, M. *et al*.^12^ involving very high incidence (26%) is excluded. Importantly, there were no statistical association between rectal NSAIDs and relevant adverse events attributable to NSAIDs use, including bleeding, perforation, ARF and cholangitis, or death events. Findings from our pooled results of 15 studies, as well as the previous published data, suggest that standard dose administration of rectal NSAIDs pre- or post-ERCP does not increase the risk of bleeding^1^. On the whole, rectal NSAIDs are effective and safe for post-ERCP prophylaxis when given as a standard dose.

Limitations of this meta-analysis must be considered. First, we found mild heterogeneity across the studies in our meta-analysis. It is not surprising given that the difference in the data source, study population, NSAIDs type, the timing of administration, and study design. However, after exclusion of heterogeneity resource, the overall estimates of prophylactic efficacy do not change substantially. Second, the characteristics of included patients, diagnostic criterion of pancreatitis as well as criterion of pancreatitis severity, definition of the risk stratification of the patients and intervention regiment varied across studies, which may influence the results, thereby limiting comparability to some extent. However, pooled results remained robust in multiple sensitivity analyses. Third, we analyzed amylase concentration (24 h post-ERCP), the risk of hyperamylasemia and pain on the basis of insufficiency sample size, of which evidence was ranked as moderate or low based on GRADE framework. Significant differences between two groups might result from type 1 error, and need to be further investigated. Ideally, large-scale RCTs should be conducted in the future to compare different NSAIDs (indomethacin versus diclofenac), different doses and timing of administration (pre-procedure versus post-procedure), to determine the best NSAID, optimal dose and timing of administration.

In conclusion, this meta-analysis provides “high quality” evidence that rectal NSAIDs appear to be a safe, simple, economical strategy, and could significantly reduce the risk of post-ERCP pancreatitis in all levels of risk populations. Therefore, rectal NSAIDs should be recommended as routine clinical use for patients who undergo ERCP without exact contraindication to NSAIDs therapy or ERCP.

## Additional Information

**How to cite this article:** Hou, Y.-C. *et al*. Efficacy and safety of rectal nonsteroidal anti-inflammatory drugs for prophylaxis against post-ERCP pancreatitis: a systematic review and meta-analysis. *Sci. Rep.* **7**, 46650; doi: 10.1038/srep46650 (2017).

**Publisher's note:** Springer Nature remains neutral with regard to jurisdictional claims in published maps and institutional affiliations.

## Supplementary Material

Supplementary Information

## Figures and Tables

**Table 1:** Basic characteristics of included studies in the meta-analysis.

Source	Text	Setting	Age (mean ± SD)	Sample size	Interventions				Indications	Severity criteria
					Suppository	Route	Dose	Timing		
Murray, 2003, Scotland	Full	Single center	I: 55 ± 15 C: 58 ± 14	220	I: Diclofenac C: Placebo	Rectal	100 mg	Immediately post-ERCP	Mainly biliary disease, SOH	NA
Sotoudehmanesh, 2007, Iran	Full	Single center	I: 58.4 ± 17.1 C: 58.1 ± 16.8	490	I: Indomethacin C: Placebo	Rectal	100 mg	Immediately pre-ERCP	Mainly biliary disease, SOD, BD stone	Cotton
Montano Loza, 2007, Mexico	Full	Multicenter	I:55.37 ± 18.0 C: 51.1 ± 17.0	150	I: Indomethacin C: Glycerin	Rectal	100 mg	2 h pre-ERCP	Suspected biliary obstruction	Ranson
Khoshbaten, 2008, Iran	Full	Single center	I: 57 ± 15 C: 60 ± 17	100	I: Diclofenac C: Placebo	Rectal	100 mg	Immediately post-ERCP	Mainly BD stone	NA
Elmunzer, 2012, U.S.	Full	Multicenter	I: 44.4 ± 13.5 C: 46.0 ± 13.1	602	I: Indomethacin C: Placebo	Rectal	100 mg	Immediately post-ERCP	Mainly suspected SOD	Cotton
Dobronte, 2012, Hungary	Full	Single center	66.8 ± 16.4	228	I: Indomethacin C: Placebo	Rectal	100 mg	10 min pre-ERCP	Not specified	Cotton
Otsuka, 2012, Japan	Full	Multicenter	I: 75 C: 72	104	I: Diclofenac C: No treatment	Rectal	50 or 25 mg	30 min pre-ERCP	Mainly billary disease	Cotton
Alabd, 2013, Sudan	Abstract	NA	NA	240	I: Diclofenac C: No treatment	Rectal	100 mg	NA	NA	NA
Dobronte, 2014, Hungary	Full	Multicenter	I: 65.66 ± 16.21 C:67.68 ± 15.56	665	I: Indomethacin C: Placebo	Rectal	100 mg	10–15 min pre-ERCP	Mainly billary disease, BD stone	Cotton
Patai, 2015, Hungary	Full	Single center	I: 66.25 (23–100) C: 64.51 (20–95)	574	I: Indomethacin C: Placebo	Rectal	100 mg	1 h pre-ERCP	Mainly billary disease, suspected SOD	Cotton
Andrade-Davila, 2015, Mexico	Full	Single center	I: 51.59 ± 18.55 C: 54.0 ± 17.85	166	I: Indomethacin C: Glycerin	Rectal	100 mg	Immediately post-ERCP	Mainly billary disease, suspected SOD, biliopancreatic tumors	Cotton
Lua, 2015, Malaysia	Full	Single center	I: 50.3 ± 17.6 C: 49.6 ± 16.8	151	I: Diclofenac C: No treatment	Rectal	100 mg	Immediately post-ERCP	Mainly billary disease	Cotton
Levenick, 2016, U.S.	Full	Single center	I: 64.9 C: 64.3	449	I: Indomethacin C: Placebo	Rectal	2 × 50 mg	During ERCP	Mainly billary disease, suspected SOD, pancreatic stricture/leak/disruption/duct stone, ampullectomy	RAC
Luo, 2016, China	Full	Multicenter	I:62 (50–72) C: 63 (50–74)	2014	I: Indomethacin C: No treatment	Rectal	100 mg	30 min pre-ERCP	Mainly billary disease, BD stone, suspected SOD	Cotton
Ucar, 2016, Turkey	Full	Single center	I: 59 ± 18.6 C: 60.5 ± 17.6	100	I: Diclofenac C: No treatment	Rectal	100 mg	30–90 min pre-ERCP	Mainly billary disease, biliopancreatic tumors	Cotton
Hosseini, 2016, Iran	Full	Single center	I: 51.2 ± 12.12 C: 49 ± 14.26	205	I: Indomethacin C: Glycerin	Rectal	100 mg	2 h pre-ERCP	Choledocolithiasis	Ranson

RAC, Revised Atlanta Classification; I, intervention; C, control; NA, not available; SOH, sphincter of Oddi hypertension; SOD, sphincter of Oddi dysfunction; BD, bile duct.

**Table 2:** Main outcome data of studies included in the meta-analysis.

Study	Severity			Amylase (mean ± SD)		Hyperamylasemia	Pain	Complications			Deaths
	Any	Mild	Moderate to severe	2 h after ERCP	24 h after ERCP			Bleeding	Perforation	Cholangitis	
Murray *et al*.	I: 7 C: 17	I: 7 C: 15	I: 0 C: 2	I: 313 ± 398.55 C: 400 ± 702.7	I: 321 ± 597.85 C: 507 ± 943.92	NA	None	None	None	None	None
Sotoudehmanesh *et al*.	I: 7 C: 15	I: 7 C: 10	I: 0 C: 5	I: 472.70** ± **92.6 C: 494.30 ± 105.2	NA	NA	None	None	None	None	None
Montano Loza *et al*.	I: 4 C: 12	None	None	I: 148.22** ± **190.60 C: 240.73** ± **256.20	NA	I: 13 C: 28	None	None	None	None	None
Khoshbaten *et al*.	I: 2 C: 13	NA	NA	I: 310.28 ± 320.62 C: 667.80 ± 1034.16	I: 324.22 ± 331.65 C: 948.86 ± 1269.69	NA	None	None	None	None	None
Elmunzer *et al*.	I: 27 C: 52	I: 14 C: 25	I: 13 C: 27	NA	NA	NA	None	I: 4 C: 7	None	None	None
Dobronte *et al*.	I: 11 C: 11	I: 9 C: 10	I: 2 C: 1	NA	NA	NA	None	None	None	None	None
Otsuka *et al*.	I: 2 C: 10	I: 2 C: 7	I: 0 C: 3	NA	NA	I: 16 C: 19	I: 4 C: 20	None	None	None	None
Alabd *et al*.	I: 4 C: 7	I: 3 C: 6	I: 1 C: 1	NA	NA	NA	None	None	None	None	None
Dobronte *et al*.	I: 20 C: 22	I: 16 C: 18	I: 4 C: 4	NA	NA	I: 81 C: 79	None	None	None	None	I: 0 C: 1
Patai *et al*.	I: 18 C: 37	I: 15 C: 33	I: 3 C: 4	NA	NA	I: 61 C: 66	None	I: 9 C: 3	I: 1 C: 0	I: 2 C: 2	I: 3 C: 4
Andrade-Davila *et al*.	I: 4 C: 17	I: 3 C: 14	I: 1 C: 3	I: 141.9** ± **92.6 C: 216.5** ± **105.2	NA	I: 19 C: 81	None	I: 2 C: 3	None	None	None
Lua *et al*.	I: 7 C: 4	I: 4 C: 4	I: 3 C: 0	NA	NA	NA	None	I: 1 C: 3	I: 1 C: 0	I: 2 C: 4	None
Levenick *et al*.	I: 16 C: 11	I: 16 C: 9	I: 0 C: 2	NA	NA	NA	None	I: 4 C: 6	I: 1 C: 0	None	I: 0 C: 3
Luo *et al*.	I: 29 C: 65	I: 22 C: 7	I: 48 C: 17	NA	NA	NA	None	NA	NA	NA	NA
Ucar *et al*.	I: 1 C: 7	I: 0 C: 3	I: 1 C: 4	NA	I: 211 ± 77 C: 463 ± 100	I: 6 C: 14	I: 5 C: 10	I: 2 C: 2	None	None	None
Hosseini *et al*.	I: 11 C: 17	None	None	NA	NA	NA	None	None	None	None	None

I, intervention; C, control; SD, standard deviation; NA, not available; ERCP, endoscopic retrograde cholangiopancreatography; ARF, acute renal failure.

**Table 3:** Summary of findings and quality of evidence assessment for the efficacy and safety of rectal NSAIDs versus no treatment in prevention of post-ERCP pancreatitis according to GRADE framework.

Main outcomes	Summary of findings			Quality of evidence assessment (GRADE)				
	No. of patients (trials)	Effect size* (95% CI)	Study limitation§	Inconsistency†	Indirectness	Imprecision‡	Quality	Importance
**Post-ERCP pancreatitis**								
Any	6458 (16)	0.55 (0.42–0.71)	None	None	None	None	High	Critical
Mild	6208 (14)	0.59 (0.48–0.73)	None	None	None	None	High	Critical
Moderate to severe	5955 (13)	0.51 (0.35–0.75)	None	None	None	None	High	Critical
**Amylase concentrations**								
2 h post-ERCP	1126 (5)	−77.85 (−104.61–51.09)	None	None	None	None	High	Important
24 h post-ERCP	420 (3)	−285.02 (−440.22–129.83)	None	−1**	None	−1¶	Low	Important
**Hyperamylasemia**	1759 (6)	0.59 (0.36–0.96)	None	−1**	None	None	Moderate	Important
**Pain**	204 (2)	0.31 (0.15–0.61)	None	None	None	−1¶	Moderate	Important
**Adverse events**								
Bleeding	4424 (15)	1.03 (0.58–1.85)	None	None	None	None	High	Critical
Perforation	4424 (15)	3.27 (0.34–31.12)	None	None	None	None	High	Critical
Cholangitis	4424 (15)	0.74 (0.21–2.59)	None	None	None	None	High	Critical
ARF	4424 (15)	0.21 (0.01–4.32)	None	None	None	None	High	Critical
Anal itching	4424 (15)	1.02 (0.15–7.10)	None	None	None	None	High	Critical
Death	4424 (15)	0.44 (0.14–1.42)	None	None	None	None	High	Critical
Total	4424 (15)	0.82 (0.51–1.31)	None	None	None	None	High	Critical

ARF, acute renal failure; ERCP, endoscopic retrograde cholangiopancreatography; GRADE, grading of recommendations assessment, development and evaluation; NSAIDs, nonsteroidal anti-inflammatory drugs. *Relative risks for post-ERCP pancreatitis, hyperamylasemia, pain and adverse events; weighted mean differences in amylase concentrations. §GRADE was downgraded by one level for the limitation of study if more than a quarter of studies included were considered at high risk of bias. †Inconsistency was considered when the heterogeneity between studies was large (*I*^*2*^ > 50%). ^‡^Imprecision was considered if few patients or few events were included in studies, and wide confidence intervals were identified around the estimate of the effect. **large heterogeneity between studies (*I*^*2*^ > 50%). ¶Low number of studies with few patients included.

**Table 4:** Subgroup analyses and sensitivity analyses of the efficacy of rectal NSAIDs versus no treatment in preventing post-ERCP pancreatitis.

Subgroup analyses						Sensitivity analyses				
Subgroup	No. of patients (trials)	Test of relationship		Test of heterogeneity		No. of patients (trials)	Test of relationship		Test of heterogeneity	
		RR (95% CI)	*P* value	*I*^*2*^, %	*P* value		RR (95% CI)	*P* value	*I*^*2*^, %	*P* value
Total	6458 (16)	0.55 (0.42–0.71)	<0.01	41	0.04	6009 (15)	0.51 (0.41–0.65)	<0.01	24	0.18
Type of NSAIDs										
Indomethacin	5543 (10)	0.58 (0.45–0.75)	<0.01	34	0.13					
Diclofenac	915 (6)	0.41 (0.19–0.90)	<0.01	55	0.05	764 (5)	0.32 (0.19–0.56)	<0.01	0	0.52
Timing of administration										
Pre-ERCP	4530 (9)	0.53 (0.42–0.67)	<0.01	0	0.43					
Post-ERCP	1239 (5)	0.46 (0.24–0.89)	<0.01	61	0.03	1088 (4)	0.39 (0.24–0.63)	<0.01	30	0.23
Population										
High risk^¶^	1135 (5)	0.41 (0.19–0.91)	0.03	66	0.02	984 (4)	0.31 (0.16–0.61)	<0.01	44	0.14
Mixed risk^**†**^	2095 (7)	0.54 (0.33–0.88)	0.01	49	0.07	1646 (6)	0.46 (0.33–0.66)	<0.01	0	0.51
Average risk^**‡**^	2884 (3)	0.60 (0.41–0.88)	<0.01	28	0.25					
Mean age										
≤60	2184 (9)	0.46 (0.31–0.69)	<0.01	39	0.11					
>60	4034 (6)	0.64 (0.43–0.96)	0.03	55	0.05	3585 (5)	0.55 (0.40–0.76)	<0.01	22	0.28
Pancreatic stent										
Yes	3702 (7)	0.56 (0.34, 0.91)	0.02	67	<0.01					
No	2516 (8)	0.56 (0.42, 0.75)	0.01	6	0.39					

NSAIDs, non-steroidal anti-inflammatory drugs; ERCP, endoscopic retrograde cholangiopancreatography; RR, relative risk; CI, confidence interval. Patients were considered as high-risk for post-ERCP pancreatitis if they met one or more of the major criteria: clinical suspicion of SOD, a history of post-ERCP pancreatitis, pancreatic sphincterotomy, precut sphincterotomy, ≥8 cannulation attempts, pneumatic dilatation of an intact biliary sphincter, or ampullectomy. Additionally, patients were also considered as high-risk for post-ERCP pancreatitis if they met at least two of the minor criteria: female less than 50 years, a history of recurrent pancreatitis (≥2 episodes), ≥3 injections of contrast agent into the pancreatic duct with ≥1 injection to the tail of the pancreas, excessive injection of contrast agent into the pancreatic duct resulting in opacification of pancreatic acini, or the acquisition of a cytologic specimen from the pancreatic duct with the use of a brush. Patients in the study by Khoshbaten *et al*. were also identified as high-risk for post-ERCP pancreatitis because they all underwent endoscopic retrograde pancreatography ± cholangiography due to extrahepatic cholestasis and/or impaired liver function tests. ^‡^Patients were considered as average-risk for post-ERCP pancreatitis if they did not meet above mentioned criteria for high-risk for post-ERCP pancreatitis. ^†^Patients (e.g. unselected patients) were considered as mixed-risk for post-ERCP pancreatitis if the criteria of risk stratification of patients in included studies were not explicitly defined.

## References

[b1] Freeman M. L. . Complications of endoscopic biliary sphincterotomy. The New England journal of medicine 335, 909–918, doi: 10.1056/nejm199609263351301 (1996). 8782497

[b2] Loperfido S. . Major early complications from diagnostic and therapeutic ERCP: a prospective multicenter study. Gastrointestinal endoscopy 48, 1–10 (1998). 9684657 10.1016/s0016-5107(98)70121-x

[b3] Li Z. S. . Effect of octreotide administration in the prophylaxis of post-ERCP pancreatitis and hyperamylasemia: A multicenter, placebo-controlled, randomized clinical trial. The American journal of gastroenterology 102, 46–51, doi: 10.1111/j.1572-0241.2006.00959.x (2007). 17266687

[b4] Fogel E. L., Eversman D., Jamidar P., Sherman S. & Lehman G. A. Sphincter of Oddi dysfunction: pancreaticobiliary sphincterotomy with pancreatic stent placement has a lower rate of pancreatitis than biliary sphincterotomy alone. Endoscopy 34, 280–285, doi: 10.1055/s-2002-23629 (2002). 11932782

[b5] Pezzilli R., Romboli E., Campana D. & Corinaldesi R. Mechanisms involved in the onset of post-ERCP pancreatitis. JOP: Journal of the pancreas 3, 162–168 (2002). 12432182

[b6] Freeman M. L. & Guda N. M. Prevention of post-ERCP pancreatitis: a comprehensive review. Gastrointestinal endoscopy 59, 845–864 (2004). 15173799 10.1016/s0016-5107(04)00353-0

[b7] Vaira D. . Endoscopic sphincterotomy in 1000 consecutive patients. Lancet (London, England) 2, 431–434 (1989). 10.1016/s0140-6736(89)90602-8 2569609

[b8] Concepcion-Martin M. . Somatostatin for prevention of post-ERCP pancreatitis: a randomized, double-blind trial. Endoscopy 46, 851–856, doi: 10.1055/s-0034-1377306 (2014). 24977398

[b9] Andriulli A. . Prophylactic administration of somatostatin or gabexate does not prevent pancreatitis after ERCP: an updated meta-analysis. Gastrointestinal endoscopy 65, 624–632, doi: 10.1016/j.gie.2006.10.030 (2007). 17383459

[b10] Wang J., Su J., Lu Y., Zhou H. & Gong B. A randomized control study to investigate the application of Ulinastatin-containing contrast medium to prevent post-ERCP pancreatitis. Hepato-gastroenterology 61, 2391–2394 (2014). 25699389

[b11] Elmunzer B. J. . A randomized trial of rectal indomethacin to prevent post-ERCP pancreatitis. The New England journal of medicine 366, 1414–1422, doi: 10.1056/NEJMoa1111103 (2012). 22494121 PMC3339271

[b12] Khoshbaten M. . Role of diclofenac in reducing post-endoscopic retrograde cholangiopancreatography pancreatitis. Journal of gastroenterology and hepatology 23, e11–16, doi: 10.1111/j.1440-1746.2007.05096.x (2008). 17683501

[b13] Manolakopoulos S. . Octreotide versus hydrocortisone versus placebo in the prevention of post-ERCP pancreatitis: a multicenter randomized controlled trial. Gastrointestinal endoscopy 55, 470–475, doi: 10.1067/mge.2002.122614 (2002). 11923756

[b14] Sethi S., Sethi N., Wadhwa V., Garud S. & Brown A. A meta-analysis on the role of rectal diclofenac and indomethacin in the prevention of post-endoscopic retrograde cholangiopancreatography pancreatitis. Pancreas 43, 190–197, doi: 10.1097/mpa.0000000000000090 (2014). 24518496

[b15] Smithline A. . Effect of prophylactic main pancreatic duct stenting on the incidence of biliary endoscopic sphincterotomy-induced pancreatitis in high-risk patients. Gastrointestinal endoscopy 39, 652–657 (1993). 8224687 10.1016/s0016-5107(93)70217-5

[b16] Mazaki T., Masuda H. & Takayama T. Prophylactic pancreatic stent placement and post-ERCP pancreatitis: a systematic review and meta-analysis. Endoscopy 42, 842–853, doi: 10.1055/s-0030-1255781 (2010). 20886403

[b17] Hosseini M., Shalchiantabrizi P., Yektaroudy K., Dadgarmoghaddam M. & Salari M. Prophylactic Effect of Rectal Indomethacin Administration, with and without Intravenous Hydration, on Development of Endoscopic Retrograde Cholangiopancreatography Pancreatitis Episodes: A Randomized Clinical Trial. Archives of Iranian medicine 19, 538–543, doi: 0161908/aim.004 (2016). 27544361

[b18] Luo H. . Routine pre-procedural rectal indometacin versus selective post-procedural rectal indometacin to prevent pancreatitis in patients undergoing endoscopic retrograde cholangiopancreatography: a multicentre, single-blinded, randomised controlled trial. Lancet (London, England) 387, 2293–2301, doi: 10.1016/s0140-6736(16)30310-5 (2016). 27133971

[b19] Döbrönte Z. . Is rectal indomethacin effective in preventing of post-endoscopic retrograde cholangiopancreatography pancreatitis? World Journal of Gastroenterology 20, 10151–10157 (2014). 25110443 10.3748/wjg.v20.i29.10151 PMC4123345

[b20] Alabd M. & Abdo A. Role of rectal nsaids in the prevention of post-ERCP pancreatitis. Journal of gastroenterology and hepatology 28, 495–496, doi: 10.1111/jgh.12363_2 (2013).

[b21] Dobronte Z., Toldy E., Mark L., Sarang K. & Lakner L. Effects of rectal indomethacin in the prevention of post-ERCP acute pancreatitis. Orvosi hetilap 153, 990–996, doi: 10.1556/oh.2012.29403 (2012). 22714033

[b22] Levenick J. M. . Rectal Indomethacin Does Not Prevent Post-ERCP Pancreatitis in Consecutive Patients. Gastroenterology 150, 911–917 (2016). 26775631 10.1053/j.gastro.2015.12.040 PMC4808426

[b23] Otsuka T. . Low-dose rectal diclofenac for prevention of post-endoscopic retrograde cholangiopancreatography pancreatitis: a randomized controlled trial. Journal of gastroenterology 47, 912–917, doi: 10.1007/s00535-012-0554-7 (2012). 22350703

[b24] Patai A., Solymosi N. & Patai Á. V. Effect of rectal indomethacin for preventing post-ERCP pancreatitis depends on difficulties of cannulation. Journal of clinical gastroenterology 49, 429–437, doi: 10.1097/MCG.0000000000000168 (2015). 25790233

[b25] Sotoudehmanesh R. . Indomethacin may reduce the incidence and severity of acute pancreatitis after ERCP. The American journal of gastroenterology 102, 978–983, doi: 10.1111/j.1572-0241.2007.01165.x (2007). 17355281

[b26] Ucar R. . Rectal or intramuscular diclofenac reduces the incidence of pancreatitis afterendoscopic retrograde cholangiopancreatography. Turkish journal of medical sciences 46, 1059–1063, doi: 10.3906/sag-1502-104 (2016). 27513404

[b27] Andrade-Davila V. F. . Rectal indomethacin versus placebo to reduce the incidence of pancreatitis after endoscopic retrograde cholangiopancreatography: results of a controlled clinical trial. BMC gastroenterology 15, 85, doi: 10.1186/s12876-015-0314-2 (2015). 26195123 PMC4508969

[b28] Lua G. W., Muthukaruppan R. & Menon J. Can Rectal Diclofenac Prevent Post Endoscopic Retrograde Cholangiopancreatography Pancreatitis? Digestive diseases and sciences 60, 3118–3123, doi: 10.1007/s10620-015-3609-9 (2015). 25757446

[b29] Murray B., Carter R., Imrie C., Evans S. & O’Suilleabhain C. Diclofenac reduces the incidence of acute pancreatitis after endoscopic retrograde cholangiopancreatography. Gastroenterology 124, 1786–1791 (2003). 12806612 10.1016/s0016-5085(03)00384-6

[b30] Yokoe M. . Japanese guidelines for the management of acute pancreatitis: Japanese Guidelines 2015. Journal of hepato-biliary-pancreatic sciences 22, 405–432, doi: 10.1002/jhbp.259 (2015). 25973947

[b31] Dumonceau J. M. . Prophylaxis of post-ERCP pancreatitis: European Society of Gastrointestinal Endoscopy (ESGE) Guideline -updated June 2014. Endoscopy 46, 799–815, doi: 10.1055/s-0034-1377875 (2014). 25148137

[b32] Higgins J. P. . The Cochrane Collaboration’s tool for assessing risk of bias in randomised trials. BMJ (Clinical research ed.) 343, d5928, doi: 10.1136/bmj.d5928 (2011). PMC3196245 22008217

[b33] Guyatt G. H. . GRADE: an emerging consensus on rating quality of evidence and strength of recommendations. BMJ (Clinical *research ed*.) 336, 924–926, doi: 10.1136/bmj.39489.470347.AD (2008). PMC2335261 18436948

[b34] Guyatt G. H. . GRADE guidelines: 2. Framing the question and deciding on important outcomes. Journal of clinical epidemiology 64, 395–400, doi: 10.1016/j.jclinepi.2010.09.012 (2011). 21194891

[b35] Balshem H. . GRADE guidelines: 3. Rating the quality of evidence. Journal of clinical epidemiology 64, 401–406, doi: 10.1016/j.jclinepi.2010.07.015 (2011). 21208779

[b36] Tonini C., Beghi E., Telaro E. & Candelise L. The Cochrane collaboration in neurology: acquisitions, research, and new initiatives. Neuroepidemiology 20, 153–159, doi: 54777 (2001). 11359086 10.1159/000054777

[b37] Robins J., Breslow N. & Greenland S. Estimators of the Mantel-Haenszel variance consistent in both sparse data and large-strata limiting models. Biometrics 42, 311–323 (1986). 3741973

[b38] DerSimonian R. & Laird N. Meta-analysis in clinical trials. Controlled clinical trials 7, 177–188 (1986). 3802833 10.1016/0197-2456(86)90046-2

[b39] Masci E., Mariani A., Curioni S. & Testoni P. A. Risk factors for pancreatitis following endoscopic retrograde cholangiopancreatography: a meta-analysis. Endoscopy 35, 830–834, doi: 10.1055/s-2003-42614 (2003). 14551860

[b40] Freeman M. L. Pancreatic stents for prevention of post-endoscopic retrograde cholangiopancreatography pancreatitis. Clinical gastroenterology and hepatology: the official clinical practice journal of the American Gastroenterological Association 5, 1354–1365, doi: 10.1016/j.cgh.2007.09.007 (2007). 17981248

[b41] Begg C. B. & Mazumdar M. Operating characteristics of a rank correlation test for publication bias. Biometrics 50, 1088–1101 (1994). 7786990

[b42] Egger M., Davey Smith G., Schneider M. & Minder C. Bias in meta-analysis detected by a simple, graphical test. BMJ (Clinical *research ed*.) 315, 629–634 (1997). 10.1136/bmj.315.7109.629 PMC2127453 9310563

[b43] Dobronte Z. . Is rectal indomethacin effective in preventing of post-endoscopic retrograde cholangiopancreatography pancreatitis? World journal of gastroenterology 20, 10151–10157, doi: 10.3748/wjg.v20.i29.10151 (2014). 25110443 PMC4123345

[b44] Montano Loza A. . [Effect of the administration of rectal indomethacin on amylase serum levels after endoscopic retrograde cholangiopancreatography, and its impact on the development of secondary pancreatitis episodes]. Revista espanola de enfermedades digestivas: organo oficial de la Sociedad Espanola de Patologia Digestiva 99, 330–336 (2007). 17883296 10.4321/s1130-01082007000600005

[b45] Dumonceau J. M. . European Society of Gastrointestinal Endoscopy (ESGE) Guideline: prophylaxis of post-ERCP pancreatitis. Endoscopy 42, 503–515, doi: 10.1055/s-0029-1244208 (2010). 20506068

[b46] Ding X., Chen M., Huang S., Zhang S. & Zou X. Nonsteroidal anti-inflammatory drugs for prevention of post-ERCP pancreatitis: a meta-analysis. Gastrointestinal endoscopy 76, 1152–1159, doi: 10.1016/j.gie.2012.08.021 (2012). 23164513

[b47] Sun H. L., Han B., Zhai H. P., Cheng X. H. & Ma K. Rectal NSAIDs for the prevention of post-ERCP pancreatitis: a meta-analysis of randomized controlled trials. The surgeon: journal of the Royal Colleges of Surgeons of Edinburgh and Ireland 12, 141–147, doi: 10.1016/j.surge.2013.10.010 (2014). 24332479

[b48] Puig I. . How and when should NSAIDs be used for preventing post-ERCP pancreatitis? A systematic review and meta-analysis. PLoS One 9, e92922, doi: 10.1371/journal.pone.0092922 (2014). 24675922 PMC3968039

[b49] Elmunzer B. J. . A meta-analysis of rectal NSAIDs in the prevention of post-ERCP pancreatitis. Gut 57, 1262–1267, doi: 10.1136/gut.2007.140756 (2008). 18375470

[b50] Inamdar S., Han D., Passi M., Sejpal D. V. & Trindade A. J. Rectal indomethacin is protective against post-ERCP pancreatitis in high-risk patients but not average-risk patients: a systematic review and meta-analysis. LID - S0016-5107(16)30540-5 [pii] LID – doi: 10.1016/j.gie.2016.08.034 (2016). 27612923

