# Supplementary material for: Efficacy and safety of rectal nonsteroidal anti-inflammatory drugs for prophylaxis against post-ERCP pancreatitis: a systematic review and meta-analysis
Source: Sci Rep. 2017 Apr 25;7:46650. doi: 10.1038/srep46650 (PMC5404221; doi:10.1038/srep46650)
Supplement: Supplementary Information [file srep46650-s1.pdf]

Efficacy and safety of rectal nonsteroidal anti-inflammatory drugs for prophylaxis against post-ERCP pancreatitis: a systematic review and meta-analysis

Yi-Chao Hou[1], Qiang Hu[1], Jiao Huang[1], Jing-Yuan Fang[1*], Hua Xiong[1*]

Appendix

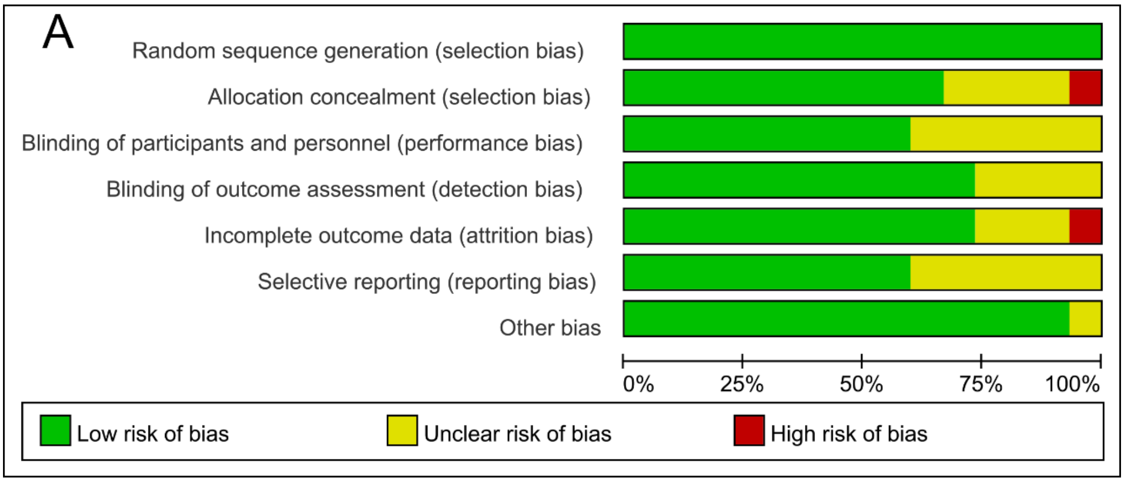

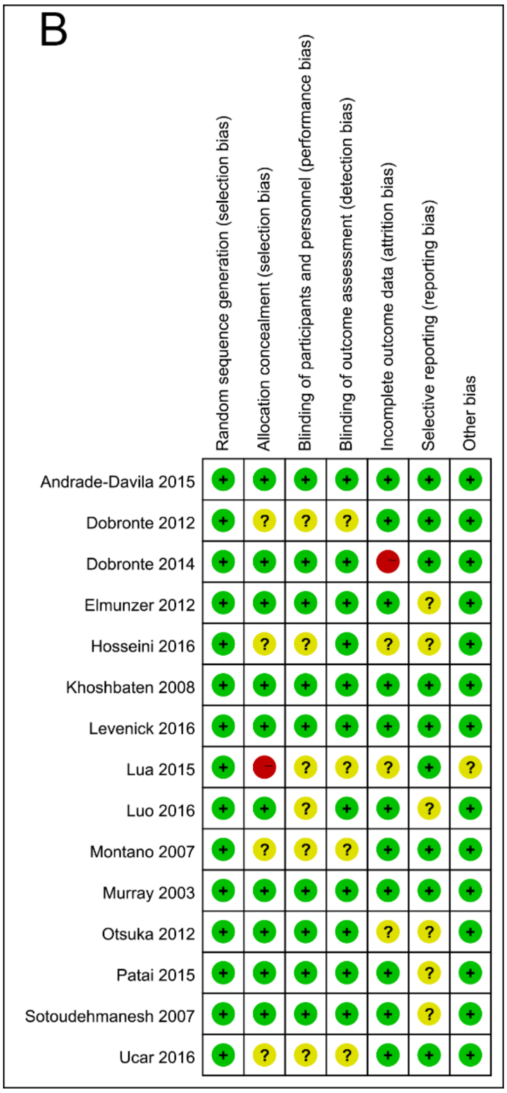

Supplementary Figure 1. The risk of bias assessments across included studies. (A) Overall risk of bias, showing review author's judgment about each risk of bias domain are presented as percentages across included studies;(B) study-level risk of bias, Green, low risk; Yellow, unclear; Red, high risk. Using Cochrane's risk of bias assessment, studies were considered to be at high, low, or unclear risk of bias based on (1) adequacy of sequence generation (2) allocation concealment (3) blinding (4) method of addressing incomplete data (5) incomplete outcome data (6) selective reporting (7) other bias.

Study or Subgroup	NSAIDs Mean	NSAIDs SD	NSAIDs Total	Control Mean	Control SD	Control Total	Weight	Mean Difference IV, Random, 95% CI
Andrade-Davila 2015	141.9	92.6	82	216.5	105.2	84	74.6%	-74.60 [-104.73, -44.47]
Khoshbaten 2008	310.28	320.62	50	667.8	1,034.16	50	1.0%	-357.52 [-657.63, -57.41]
Montano Loza 2007	148.22	190.6	75	240.73	256.2	75	16.2%	-92.51 [-164.78, -20.24]
Murray 2003	313	398.55	110	400	702.7	110	3.9%	-87.00 [-237.97, 63.97]
Sotoudehmanesh 2007	472.7	910.4	245	494.3	694.1	245	4.3%	-21.60 [-164.95, 121.75]
**Total (95% CI)**			**562**			**564**	**100.0%**	**-78.51 [-108.41, -48.61]**

Heterogeneity: Tau² = 75.40; Chi² = 4.14, df = 4 (P = 0.39); I² = 3%
Test for overall effect: Z = 5.15 (P < 0.00001)

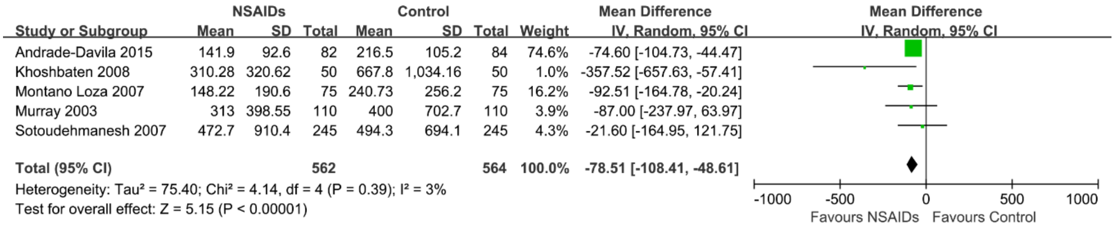

Supplementary Figure 2. Forest plot showing a significant reduction in the serum amylase level 2 h post-ERCP with rectal NSAIDs therapy. IV, IV, inverse variance; SE, standard error; NSAIDs, nonsteroidal anti-inflammatory drugs; ERCP, endoscopic retrograde cholangiopancreatography.

Study or Subgroup	NSAIDs Mean	NSAIDs SD	NSAIDs Total	Control Mean	Control SD	Control Total	Weight	Mean Difference IV, Random, 95% CI
Khoshbaten 2008	324.22	331.65	50	948.86	1,269.69	50	13.9%	-624.64 [-988.38, -260.90]
Murray 2003	321	597.85	110	507	943.92	110	28.6%	-186.00 [-394.80, 22.80]
Ucar 2016	211	77	50	463	100	50	57.5%	-252.00 [-286.98, -217.02]
**Total (95% CI)**			**210**			**210**	**100.0%**	**-285.02 [-440.22, -129.83]**

Heterogeneity: Tau² = 10586.94; Chi² = 4.41, df = 2 (P = 0.11); I² = 55%

Test for overall effect: Z = 3.60 (P = 0.0003)

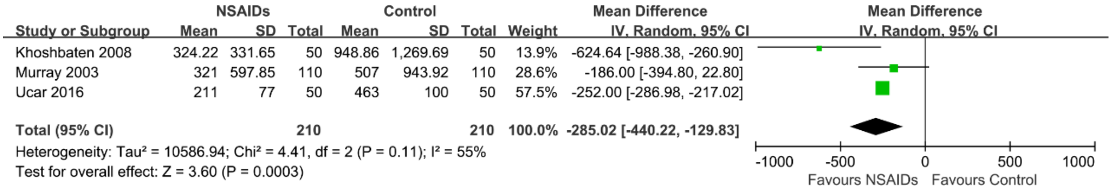

Supplementary Figure 3. Forest plot showing a significant reduction in the serum amylase level 24 h post-ERCP with rectal NSAIDs therapy. IV, IV, inverse variance; SE, standard error; NSAIDs, nonsteroidal anti-inflammatory drugs; ERCP, endoscopic retrograde cholangiopancreatography.

Study or Subgroup	NSAIDS Events	NSAIDS Total	Control Events	Control Total	Weight	Risk Ratio M-H, Random, 95% CI
Andrade-Davila 2015	19	82	81	84	17.9%	0.24 [0.16, 0.36]
Dobronte 2014	81	347	79	318	19.0%	0.94 [0.72, 1.23]
Montano Loza 2007	13	75	28	75	15.8%	0.46 [0.26, 0.82]
Otsuka 2012	16	51	19	53	16.2%	0.88 [0.51, 1.51]
Patai 2015	61	287	66	287	18.7%	0.92 [0.68, 1.26]
Ucar 2016	6	50	14	50	12.3%	0.43 [0.18, 1.03]
**Total (95% CI)**		**892**		**867**	**100.0%**	**0.59 [0.36, 0.96]**
Total events	196		287			

Heterogeneity: Tau² = 0.31; Chi² = 39.49, df = 5 (P < 0.00001); I² = 87%
Test for overall effect: Z = 2.11 (P = 0.03)

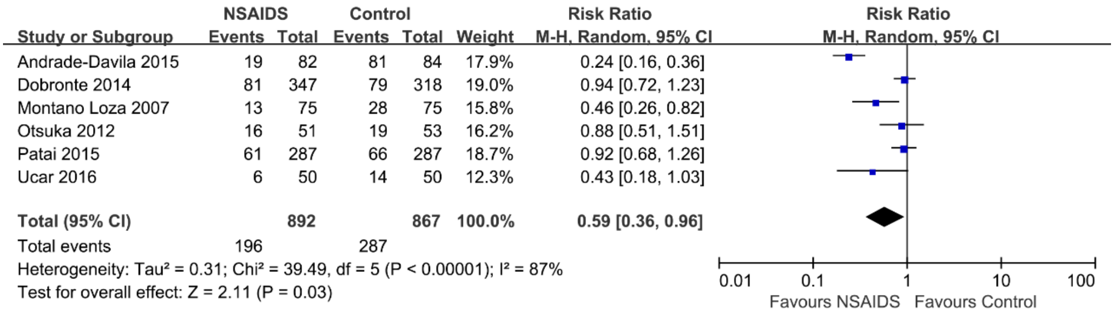

Supplementary Figure 4. Forest plot showing a significant reduction in the risk of hyperamylasemia with rectal NSAIDs therapy. M-H, Mantel-Haenszel; SE, standard error; NSAIDs, nonsteroidal anti-inflammatory drugs.

Study or Subgroup	NSAIDs Events	NSAIDs Total	Control Events	Control Total	Weight	Risk Ratio M-H, Random, 95% CI
Otsuka 2012	4	51	20	53	49.9%	0.21 [0.08, 0.57]
Ucar 2016	5	50	10	50	50.1%	0.50 [0.18, 1.36]
**Total (95% CI)**		**101**		**103**	**100.0%**	**0.32 [0.14, 0.77]**
Total events	9		30			

Heterogeneity: Tau² = 0.13; Chi² = 1.50, df = 1 (P = 0.22); I² = 33%
Test for overall effect: Z = 2.56 (P = 0.01)

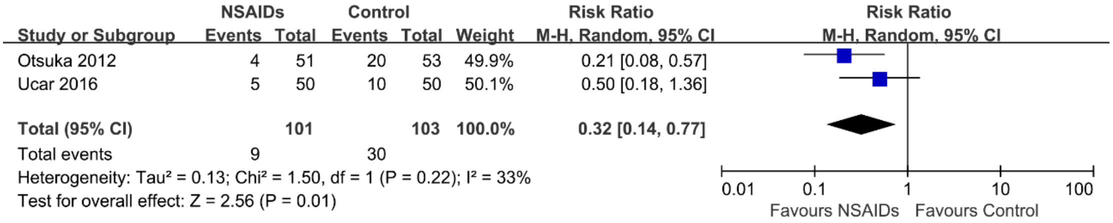

Supplementary Figure 5. Forest plot showing a significant reduction in the risk of pain with rectal NSAIDs therapy. M-H, Mantel-Haenszel; SE, standard error; NSAIDs, nonsteroidal anti-inflammatory drugs.

PubMed

No.	Query Results	Results	Date
#1.	"Anti-Inflammatory Agents, Non-Steroidal"[Mesh]	72381	09 Aug 2016
#2.	"diclofenac" [Title/Abstract] OR "indomethacin" [Title/Abstract] OR "aspirin" [Title/Abstract] OR "ibuprofen" [Title/Abstract] OR "naproxen" [Title/Abstract] OR "ketorolac" [Title/Abstract] OR "etodolac" [Title/Abstract] OR "sulindac" [Title/Abstract] OR "COX-2 inhibitors" [Title/Abstract] OR "celecoxib" [Title/Abstract] OR "rofecoxib" [Title/Abstract] OR "valdecoxib" [Title/Abstract]	102230	09 Aug 2016
#3.	"diclofenac" [Title/Abstract] OR "indomethacin" [Title/Abstract] OR "aspirin" [Title/Abstract] OR "ibuprofen" [Title/Abstract] OR "naproxen" [Title/Abstract] OR "ketorolac" [Title/Abstract] OR "etodolac" [Title/Abstract] OR "sulindac" [Title/Abstract] OR "COX-2 inhibitors" [Title/Abstract] OR "celecoxib" [Title/Abstract] OR "rofecoxib" [Title/Abstract] OR "valdecoxib" [Title/Abstract] OR "Anti-Inflammatory Agents, Non-Steroidal"[Mesh]	148093	09 Aug 2016
#4.	"Pancreatitis"[Mesh]	46195	09 Aug 2016
#5.	"post-ERCP pancreatitis" [Title/Abstract]) OR "post-endoscopic retrograde cholangiopancreatography pancreatitis" [Title/Abstract]	791	09 Aug 2016
#6.	"Pancreatitis"[Mesh]) OR "post-ERCP pancreatitis" [Title/Abstract] OR "post-endoscopic retrograde cholangiopancreatography pancreatitis" [Title/Abstract]	46453	09 Aug 2016
#7.	#3 and #6	362	09 Aug 2016

EMBASE

No.	Query Results	Results	Date
#1.	'nonsteroid antiinflammatory agent'/exp	497,744	09 Aug 2016
#2.	'diclofenac':ab,ti OR 'indomethacin':ab,ti OR 'aspirin':ab,ti OR 'ibuprofen':ab,ti OR 'naproxen':ab,ti OR 'ketorolac':ab,ti OR 'etodolac':ab,ti OR 'sulindac':ab,ti OR 'cox-2 inhibitors':ab,ti OR 'celecoxib':ab,ti OR 'rofecoxib':ab,ti OR 'valdecoxib':ab,ti	137799	09 Aug 2016
#3.	'nonsteroid antiinflammatory agent'/exp OR 'diclofenac':ab,ti OR 'indomethacin':ab,ti OR 'aspirin':ab,ti OR 'ibuprofen':ab,ti OR 'naproxen':ab,ti OR 'ketorolac':ab,ti OR 'etodolac':ab,ti OR 'sulindac':ab,ti OR 'cox-2 inhibitors':ab,ti OR 'celecoxib':ab,ti OR 'rofecoxib':ab,ti OR 'valdecoxib':ab,ti	510,407	09 Aug 2016
#4.	'pancreatitis'/exp	85,885	09 Aug 2016
#5.	'post-ercp pancreatitis':ab,ti OR 'post-endoscopic retrograde cholangiopancreatography pancreatitis':ab,ti	1587	09 Aug 2016
#6.	pancreatitis'/exp OR 'post-endoscopic retrograde cholangiopancreatography pancreatitis':ab,ti OR 'post-ercp pancreatitis':ab,ti	85925	09 Aug 2016
#7.	#3 and #6	3,093	09 Aug 2016

Cochrane Library

No.	Query Results	Results	Date

#1.	MeSH descriptor: [Anti-Inflammatory Agents, Non-Steroidal] explode all trees	7068	09 Aug 2016
#2.	diclofenac:ti,ab,kw or indomethacin:ti,ab,kw or aspirin:ti,ab,kw or ibuprofen:ti,ab,kw or naproxen:ti,ab,kw	18322	09 Aug 2016
#3.	ketorolac:ti,ab,kw or etodolac:ti,ab,kw or sulindac:ti,ab,kw or COX-2 inhibitors:ti,ab,kw or celecoxib:ti,ab,kw	3667	09 Aug 2016
#4.	rofecoxib:ti,ab,kw or valdecoxib:ti,ab,kw	559	09 Aug 2016
#5.	#1 or #2 or #3 or #4	24135	
#5.	MeSH descriptor: [Pancreatitis] explode all trees	1105	09 Aug 2016
#6.	post-endoscopic retrograde cholangiopancreatography pancreatitis:ti,ab,kw or post-ERCP pancreatitis:ti,ab,kw	345	09 Aug 2016
#7.	#5 or #6	1268	09 Aug 2016
#8.	#5 and #7	82	09 Aug 2016

Supplementary Table 1. Search strategy used in PubMed, EMBASE and the Cochrane Library.
